# A giant adrenal pseudocyst presenting with right hypochondralgia and fever: a case report

**DOI:** 10.1186/1752-1947-5-135

**Published:** 2011-04-04

**Authors:** Masashi Momiyama, Kenichi Matsuo, Kenichi Yoshida, Kuniya Tanaka, Hirotoshi Akiyama, Shoji Yamanaka, Itaru Endo

**Affiliations:** 1 Department of Gastroenterological Surgery, Yokohama City University, Yokohama, Japan; 2 Division of Anatomic and Surgical Pathology, Yokohama City University Hospital, Yokohama, Japan

## Abstract

**Introduction:**

Adrenal pseudocysts are rare cystic masses that arise from the adrenal gland and which are usually non-functional and asymptomatic. Adrenal pseudocysts consist of a fibrous wall without an epithelial or endothelial lining. We report the case of a patient with a giant adrenal pseudocyst presenting with right hypochondralgia and high fever.

**Case presentation:**

A 52-year-old Japanese man was admitted with right hypochondralgia and a chill. Abdominal computed tomography revealed a well-defined cystic mass measuring 19 cm which was located in the right adrenal region and the contents of which were not enhanced with contrast medium. Abdominal ultrasonography revealed a heterogeneously hypo-echoic lesion with a peripheral high-echoic rim. Serum hormonal levels were almost normal. Despite treatment with antibiotics, the high fever persisted. Based on these findings, we made a preoperative diagnosis of a right adrenal cyst with infection. However, the possibility of malignancy still remained. The patient underwent laparotomy and right adrenal cyst excision with partial hepatectomy in order to relieve the symptoms and to confirm an accurate diagnosis. Histological examination revealed an adrenal pseudocyst with infection. His condition improved soon after the operation.

**Conclusion:**

We report a case of a giant adrenal pseudocyst with infection. Surgery is required for symptomatic cases in order to relieve the symptoms and in cases of uncertain diagnosis.

## Introduction

In 1903, Doran attributed the first case of adrenal cyst to Greiselius [1]. In 1670, he described a 45-year-old man whose death resulted from a rupture of the cyst. There were only seven cases of adrenal cyst reported by 1906. Wahl questioned the rarity of adrenal cysts in 1951 and found an autopsy incidence of 1 in 1555 [2]. The paucity of reports in the literature was a manifestation of clinical silence rather than true rarity. In 1966, Foster described 220 cases of adrenal cyst in the world's literature [3], while in 1979 Incze *et al*. reported 250 cases [4].

Cystic lesions of the adrenal gland are uncommon and demonstrate a spectrum of histological changes and may vary from pseudocysts to malignant cystic neoplasms. An adrenal pseudocyst is a fibrous-surrounded cyst within the adrenal gland devoid of a recognizable layer of lining cells. The incidence of adrenal pseudocyst with infection is very rare. Only a few cases have been found in the MEDLINE database search with 'adrenal pseudocyst' and 'infection' so far. We report a case of giant adrenal pseudocyst presenting with a right hypochondralgia and high fever, which was diagnosed as an adrenal pseudocyst with infection measuring about 19 cm in largest diameter.

## Case presentation

**Figure 1:**
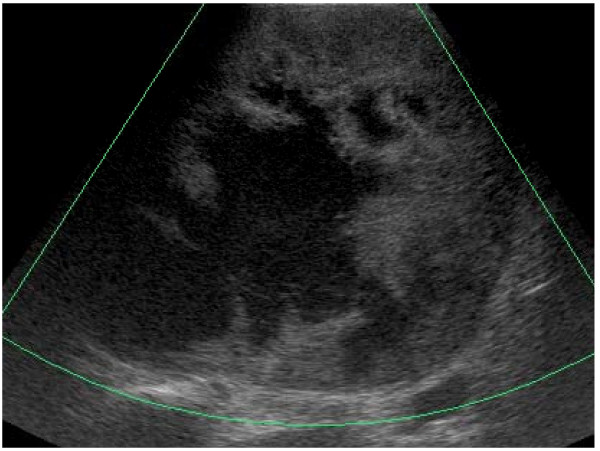
**Abdominal ultrasonography revealed an 18 × 18 cm, heterogeneously hypo-echoic lesion in the right adrenal area with peripheral high-echoic rim**.

**Figure 2:**
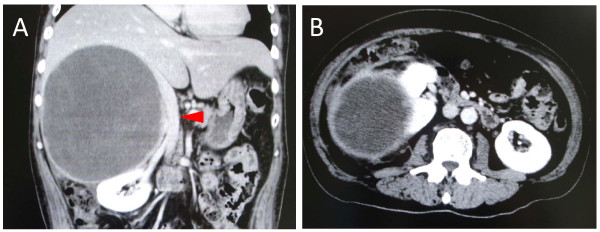
**An enhanced computed tomography of the abdomen revealed a giant homogeneous low density mass lesion in the right adrenal region**. (A) A giant mass lesion was indented over the inferior aspect of the right lobe of the liver and a displaced inferior vena cava (arrowhead) with no abdominal lymphadenopathy. (B) The right kidney was also ventrally displaced.

**Figure 3:**
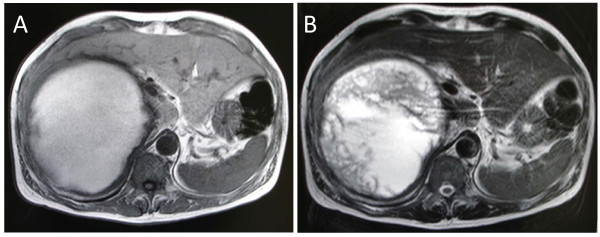
**Abdominal magnetic resonance imaging revealed a well-defined high intensity mass which appeared homogeneous intense in the T 1-weighted image (A) but heterogeneously intense in the T 2-weighted image (B)**.

A 52-year-old Japanese man, who had an intra-abdominal cystic mass, was followed up every year in another hospital. His previous ultrasonography (US) which was performed seven years ago, revealed a unilobulated cyst, measuring 14 cm in diameter, adjacent to the liver. The internal structure of the cyst was homogeneous and there was no septation. Upon this finding his lesion was misdiagnosed as a liver cyst and it was suggested that it be monitored. He was admitted to our hospital with a two-month history of right hypochondralgia and high fever. On clinical examination, he was febrile with temperature of 38.0°C. His blood pressure was 116/76 mm Hg without orthostatic changes or tachycardia. A clearly defined mass occupied the right hypochondrium and was tender. Laboratory investigation showed: a total leukocyte count of 12,200/mm^3^; C-reactive protein of 23.7 mg/dL; alkaline phosphatase of 634 U/L; and gamma glutamyl transpeptidase of 183 U/L. The hormonal examination, serum catecholamines, cortisol and aldosterone were all within normal limits. Fasting blood sugar, renal functions and liver function were within normal limits. Tumor marker (carcinoembryonic antigen, carbohydrate antigen 19-9 and alpha-fetoprotein) levels were within normal limits; urine and stool examination, chest X-ray, gastrointestinal endoscopy and colonoscopy did not reveal any abnormalities. He underwent several imaging investigations. An abdominal US revealed an 18 × 18 cm heterogeneously hypoechoic lesion in the right adrenal area with a peripheral highechoic rim (Figure 1). An enhanced computed tomography (CT) of the abdomen revealed a giant homogeneous low density mass lesion in the right adrenal region indenting over the inferior aspect of the right lobe of the liver, displacing the inferior vena cava with no abdominal lymphadenopathy (Figure 2a). The content of the lesion was not enhanced with contrast medium. His right kidney was also ventrally displaced (Figure 2b). Clear margins between the mass and the liver could not be defined in the CT. Magnetic resonance imaging (MRI) revealed a well-defined high intensity mass which appeared homogeneous intense in the T1-weighted image (Figure 3a) but heterogeneous intense in the T2-weighted image (Figure 3b). Based on these findings, the patient was diagnosed as having an adrenal cyst with infection.

**Figure 4:**
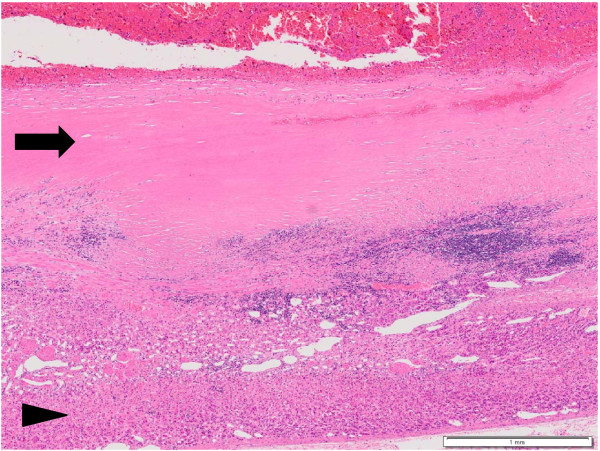
**Histological examination of the cyst showed an adrenal pseudocyst**. The cystic wall consisted of dense fibrous tissue without an epithelial lining (arrow). A rim of normal adrenal tissue was found compressed within the cystic capsule (arrow head). (hematoxylin and eosin stain, Scale bar = 1 mm).

After admission, treatment with antibiotics (imipenem/cilastatin sodium) was started. Despite treatment with antibiotics, the high fever persisted. He had blood culture taken three times during a fever episode and all of the results were negative. Percutaneous aspiration was not performed because of the possibility of its malignancy due to the large size of the mass. The patient underwent reverse-L-type laparotomy and excision of the right adrenal cyst. At laparotomy, the cyst was found to be densely adhered to the posterior abdominal wall, the liver, the inferior vena cava and the right kidney. It was resected concomitant with partial hepatectomy. The cyst was unilocular, measured 19 × 18 × 19 cm and weighed 1525 g. It contained a reddish brown fluid and a culture of the fluid showed *Staphylococcus captis*. The histological examination showed that the cystic wall was 0.6 cm to 1.1 cm thick and consisted of dense fibrous tissue, without an epithelial lining (Figure 4). There were areas of abscess and chronic inflammation within the fibrous tissue. A rim of the normal adrenal tissue was found to be compressed within the cystic capsule and a diagnosis of an adrenal pseudocyst was made. The patient's postoperative course was uneventful and he was discharged 10 days after the operation. The right hypochondralgia and high fever resolved after the removal of the pseudocyst.

## Discussion

Adrenal cysts are rare and the documented incidence varies between 0.064% and 0.18% in autopsy series [5]. However, the rate of detection of adrenal cysts has risen dramatically due to the more frequent use of CT and MRI imaging studies in recent years, which account for approximately 5% of incidentally discovered adrenal lesions [6]. Adrenal cysts may occur at any age but most are found in the 3rd to 5th decades [3]. In some series, a female preponderance of about 3:1 has been noted for unknown reasons [7].

Histologically, cystic formations of the adrenals are divided into four groups: parasitic; epithelial (true cysts); endothelial (vascular cysts with an endothelial lining); and pseudocysts [8]. There are also other more infrequent subtypes such as lymphangiomas, mesothelial cysts, dermoid cysts or cystic adrenal carcinomas. Adrenal pseudocysts represent approximately 80% of cystic adrenal masses [9,10]. Adrenal pseudocysts are devoid of an epithelial or endothelial lining, arise within the adrenal gland and are surrounded by a fibrous tissue wall.

The true origin of adrenal pseudocyst remains a mystery. One theory suggests that these lesions result from an intra-adrenal hemorrhage caused by trauma, a sepsis event or some other form of shock [11]. The initial injury leads to the development of a cavity with a scarred, fibrous lining that slowly enlarges over time. Another theory suggests that these lesions are true cysts that have lost their cellular lining because of the inflammation and bleeding within the cyst. The etiology of our patient's pseudocyst seemed to be similar to latter theory. The patient's lesion was diagnosed as a true cyst at first because of its homogeneity on the US finding. The internal structure of the cyst changed into heterogeneous and, finally, the cyst was diagnosed as pseudocyst.

Most adrenal cysts are asymptomatic because of their small size [3]. In the case of large cysts, symptoms occur in relation to their compression of adjacent organs. This seems to be a common feature in most pseudocysts (either they arise from the adrenals or from the pancreas) and seems to be related to the chronically increased intra-abdominal pressure that these cyst introduce [12]. The three most prominent clinical features are: a dull pain in the adrenal area; gastrointestinal symptoms; and a palpable mass. They seldom cause adrenal hypofunction, Cushing's syndrome or pheochromocytoma [8]. Acute abdomen or a tender mass may occasionally be found, when intracystic hemorrhage, rupture or infection occurs [8]. Our patient had a right hypochondralgia with tenderness and high fever due to infection, with no gastrointestinal complaints. He had no hypertension during the follow-up period.

Due to the wide use of the diagnostic imaging modalities, the detection rate of adrenal cystic lesions is increasing. However, a preoperative confirmatory diagnosis of a large adrenal cyst can be very difficult because of the indistinct boundary with surrounding organs and adhesion to neighboring organs. Furthermore, even with integrated fluorine-18 fluorodeoxyglucose positron emission tomography (PET), adrenal lesions may be identified as false-positive at PET, including adrenal adenomas, adrenal endothelial cysts and inflammatory and infectious lesions [13].

The differential diagnosis of adrenal pseudocysts includes splenic, hepatic and renal cysts, as well as mesenteric or retroperitoneal cysts, urachal cysts and solid adrenal tumors. An exact diagnosis is clinically important in large lesion because adrenal incidentalomas larger than 5 cm [14] carry an increased risk of adrenal malignancy. The reported incidence of malignancy in adrenal cystic lesions is approximately 7% [14].

On CT, most pseudocysts demonstrate well-demarcated round or oval masses with fluid density but the CT features of pseudocysts are more complicated than simple cysts due to the complicated components such as septa, blood and soft-tissue components. The cysts wall shows occasional calcification. MRI is the best modality for visualizing the complicated intracystic components. Moreover, MRI is particularly sensitive for detecting intracystic hemorrhage, which shows hyperintense on both T1- and T2-weighted images.

Treatment of adrenal cysts is determined by size and the symptoms related to the mass. Surgical excision is indicated by the presence of symptoms, a suspicion of malignancy and an increase in size, or the detection of, a functioning adrenal cyst. Surgical treatment may not be necessary for small asymptomatic lesions as most cysts are benign [14]. If the adrenal lesion is diagnosed as a simple nonfunctioning cyst, the patient may be treated conservatively with aspiration alone. In large abscesses, where the probability of rupture is increased, transcutaneous drainage should be avoided as it may increase the risk of microbial load dissemination [15].

## Conclusion

An adrenal pseudocyst is an uncommon clinical finding and is even rarer when it is giant-sized and infected. Surgery is required for symptomatic cases in order to relieve the symptoms and in cases of uncertain diagnosis. Radiological and clinical features of the tumor are nonspecific, thus, histopathological examination is essential in order to establish a definitive diagnosis.

## Abbreviations

CT: computed tomography; MRI: magnetic resonance imaging; PET: positron emission tomography; US: ultrasound.

## Competing interests

The authors declare that they have no competing interests.

## Consent

Written informed consent was obtained from the patient for publication of this case report and any accompanying images. A copy of the written consent is available for review by the Editor-in-Chief of this journal.

## Authors' contributions

MM, KM and KY were involved in drafting the manuscript. KT, HA, SY and IE revised the manuscript. All authors have read and approved the final manuscript.
